# Brief Observations on a French Work Lately Published by Dr. Mahon, Respecting Legal Medicine, &c.

**Published:** 1803-01-01

**Authors:** 

**Affiliations:** St. Pancras, Middlesex


### *Brief Observations on a French Work lately published by* Dr. Mahon, *respecting Legal Medicine, &c. communicated by* Dr. Hall, *of St. Pancras, Middlesex.*

The subject of Legal Medicine involves the consideration of those cases, in which the evidence, testimony, or opinion of medical practitioners may be required in a court of judicature. As this branch of jurisprudence is intimately intimately connected with some of the most important concerns of man in every civilized community, it must be allowed, that any attempt to afford instruction, and enlarge the boundaries of our knowledge on a subject of so much importance, cannot fail to prove generally interesting; particularly when it is considered, that many of the doctrines advanced by the earlier writers on forensic medicine, have beome, from the advanced state of science, altogether obsolete and unsatisfactory,

Some late writers, indeed, and, among others, Dr. Johnstone of Birmingham, availing themselves of the recent discoveries in chemistry, have given us much valuable information on particular branches in this department of medicine; but, as these publications embrace only a very small part of medical jurisprudence, a more extensive treatise on this subject was certainly a desideratum in English literature, and which the work under consideration seems, in a great measure, well calculated to supply; for although a small portion of the reasoning, contained in these volumes, may be inapplicable to the criminal law of this country, yet, on the whole, we may venture to affirm, that whilst Dr. Mahon has conducted his inquiries with much ingenuity and discernment, he has furnished a very considerable share of practical information, on a greater variety of subjects, connected with legal medicine, than is to be found in any other work hitherto presented to the public.

The volumes before us, commence with a general history of legal medicine; into any detail of which, we shall not here enter, since, from the present state of knowledge in this island, it could prove very little interesting to the English reader.

In this part of the work, we are presented with several humiliating examples of the absurd credulity which still prevails among professional men in some more remote parts of the Continent, as well as in France; and which our author observes, would have produced bloody scenes, when the tribunals were less enlightened.

The subject of *Impotence* is examined in a very comprehensive and satisfactory manner; but as charges on such a ground are now seldom or never brought forward in an English court of justice, we shall not enlarge on this or similar topics, in order to leave room for the consideration of others, which are of much more general importance.

In the chapter on *Hermaphrodites*, whose existence has been called in question by some naturalists, we find an interesting interesting and accurate account of the dissection of a subject, which appeared to combine the genital parts of both sexes more perfectly than any other yet described; although, even in this case, the different sexual organs were by no means complete.

Dr. M. appears to us very judiciously to attach little importance to the opinions generally entertained on the subject of *Defloration*; whilst, on that of *Rape*, we are inclined to think, he carries his incredulity to an unreasonable length. Every medical practitioner must be aware, that much attention is frequently necessary, in order to discriminate between cases of spurious or false, and those of real pregnancy. Our author's observations on this *subject* are copious and correct, and cannot fail to assist in guarding the judgment against those fallacious appearances, which sometimes not only deceive the uninstructed patient, but even the intelligent physician. After enumerating the signs from which parturition may be inferred to have taken place, he justly remarks, that in order to form a certain conclusion on the subject, they ought to be considered collectively, and within a very short period after delivery. The author next enters, at some length, into the so much agitated question respecting the protraction of gestation beyond the usual period, and combats, with no inconsiderable share of success, many of the erroneous notions formerly prevalent on the Continent, respecting this subject.

On *Abortion*, and the marks of premature birth, we also find several useful observations, but none of sufficient importance to merit particular notice. Respecting *Monsters*, it is humanely observed, that they cannot be deprived of life without a crime; and that although the general welfare of society may render it necessary to preclude them from public employments, and even sometimes from entering into the married state, yet they are nevertheless entitled to share the means of subsistence, and the protection of the laws.

*Mental Derangement* may, perhaps, be justly regarded as one of the most important topics which Dr. M. has undertaken to discuss, in the present volume. We shall not here enter into any examination of the theory advanced by him, respecting the nature of this disease, since we consider it wholly unnecessary in a judicial point of view; neither shall we follow our author in his enumeration of the diagnostic signs and appearances, which, in his opinion, characterise the presence of insanity, as they do not not appear to us very materially to differ from those given by former writers on this subject. We may, however, be permitted to remark, that, in this detail, he neither notices the protruded and glistening eye, the peculiar cast of countenance so observable in most maniacal patients, nor yet their extreme insensibility to cold, hunger, and fatigue.

Dr. M. very properly cautions his readers not to confound with mania, those states of delirium which proceed from fever, or other temporary causes; and, on the contrary, to be aware that real maniacs frequently give very pertinent answers to any questions proposed to them, and converse rationally on every subject, but on that which more immediately operated to produce the disease. Those alienations of mind, which proceed from the effect of poisonous substances, or intoxicating liquors taken into the stomach, are accurately discriminated.

In estimating the probability, whether a maniacal paroxysm actually prevailed during the commission of a crime, the author observes, that when individuals have acted in direct opposition to the common principles and feelings of mankind, both humanity and justice require us to regulate our decisions in conformity with that maxim of enlightened Jurisconsults, *semel furiosus semper presumitur furiosus, et contrarium tenenti incumbit onus probandi sanam mentem.*

Dr. M. now proceeds to the consideration of *Feigned* and *Concealed Diseases*; on which subjects, the practitioner will meet with many remarks worthy of attention. A number of facts, from different authors, are cited, and one in particular related, from his own knowledge, to evince how far frauds of this nature may sometimes be carried. As these impositions, however, may be various, no determined directions can be given, which will suit every case; much, it is therefore evident, must be left to the skill and sagacity of the physician.

In the second volume, Dr. M. enters largely into the consideration of wounds in general; but, as much of his reasoning on this subject is only applicable to the complicated principles of foreign criminal codes, we shall not follow him in this investigation. The comparative danger of wounds, in different parts of the body, are next examined with much minuteness of detail; and the combination of circumstances under which those that are not necessarily mortal, may accidentally prove fatal, are stated with much accuracy and precision. In the chapter on

*Apparent*

*Apparent Death,* we find some humane cautions against premature interment. On this subject, however, it seems here unnecessary to enlarge, as fortunately, the practice, which almost universally prevails in this country, of keeping the body unburied during several days, secures us against the possibility of any such abuse. We shall only remark, in general, that as the vital principle frequently remains in a latent state for some time, and as we are yet unacquainted with any certain criterion between positive and apparent death, besides that of putrefaction, some appearances of incipient decomposition should therefore be allowed to take place, in every case, before the period of interment.

Several pertinent remarks next follow on the danger of precipitate dissections. Such dreadful mistakes, we learn with astonishment, have oftener than once occurred within these few years in France.

A short chapter succeeds, on the subject of *Violent Death,* the principal signs of which, according to our author, are

1. Hæmorrhage.
2. Ecchymosis.
3. Inflammation.
4. Congestions of blood.
5. Every mark from which it may be presumed that the patient had suffered pain.
6. Spasms which continue even after death.

Having considered the evidence which may be drawn from each of these signs separately taken, he concludes by properly observing, that as several of these appearances may be occasioned by preceding disease, it is only from an accurate comparison of the whole, or the greatest number of them, that any certain conclusion can be formed on the subject. The next section of the present work, treats on the *Opening of Dead Bodies.* Here the practitioner, who may be called upon by the civil magistrate to perform this office, will meet with many remarks and observations highly worthy of attention.

The subject on which our author next enters, is of considerable importance, as it involves the consideration of the effects of poisonous substances on animal bodies. After giving a general enumeration of the symptoms produced by poisons on the living system, he proceeds to describe the signs which usually appear on the examination of the dead subject. He agrees with Hebenstreit, in regarding the the separation of the villous coat of the stomach as affording the most certain criterion of the exhibition of poison. This appearance, however, we can by no means consider as decisive in every case of deleterious substances being taken into the stomach, since most of the vegetable poisons act rather by an impression on the nervous system than by exciting inflammation in the stomach and bowels.

In treating of poisons in general, the author observes, "If a person in perfect health, immediately after having taken any food, drink, or medicine, finds himself suddenly attacked by vertigo, pains in the stomach, colic, vomiting, cholera morbus, spasms, convulsions, faintings, stupor, and swellings of the lips, tongue, throat, stomach, or belly, with a painful sense of burning heat; and if, in addition to these symptoms, there be found in the matter evacuated or rejected by the patient, any chewed herbs, remains of roots, mushrooms, juices, powders, salts, or pills; if he complain of a disagreeable smell or taste in the matter vomited; or, lastly, if no epidemic disease prevail, accompanied with similar symptoms, we may reasonably suspect the agency of poison. He distinguishes the equivocal from the certain symptoms here detailed, and observes, that every sign of poison may sometimes be occasioned by the patient having eaten food difficult of digestion, or in a corrupted state; mushrooms, for example, though generally reputed harmless, have frequently produced this effect, as well as a variety of shell-fish, &c. and the author himself once witnessed every sign of poison supervene upon swallowing a roasted chesnut. He likewise discriminates between the effects of acrid and irritating poisons, which destroy by producing inflammation of the stomach and bowels, and those of narcotic substances, which impair and ultimately extinguish the energy of the brain and nervous system.

The consideration of individual poisons forms the subject of a separate chapter, and, as containing many valuable observations, well deserves to be consulted, particularly by the younger part of the profession, or by those who have not made this department of medicine their peculiar study. The intricate and much agitated question of *Child-Murder* is next examined by Dr. M. with great care and attention. He combats the opinion, maintained by some writers, that the death of the child necessarily follows from the neglect of tying the umbilical cord after delivery, and shows with what limitation this doctrine should be received.

He likewise enters largely into the consideration of the proof of infanticide, deduced from the specific gravity of the the lungs, and infers, from a variety of experiments, that the pulmonary organs of a fœtus, which had never respired, will, nevertheless, swim and continue buoyant on the surface of water when putrefaction begins to take place; but, that if either the slightest pressure be employed, or when the putrefactive process has proceeded to a greater length, they will sink and remain at the bottom.

The experiments of M. Ploucquet, undertaken with the view of determining, by means of the balance, the quantity of blood introduced into the lungs by respiration, are also noticed. This gentleman weighed the body of a dead-born child, which had exhibited signs of life a few hours before delivery, and found that its total weight was 53,040 grains. The weight of the lungs, separately taken, amounted to 792 grains. Hence it appears, that the weight of the body, relatively to that of the lungs, was nearly as 67 to 1. The result of a second experiment, made on a full grown fœtus, under similar circumstances, was 70 to 1; whilst, on the contrary, in a third that had respired, although not even arrived at its full maturity, the weight of the body to that of the lungs was 70 to 2.

From these facts, M. Ploucquet infers, that the weight of the lungs is doubled by the introduction of blood into that organ, in consequence of respiration; and that, in doubtful cases, this great augmentation of weight will furnish a certain criterion by which we may distinguish between a fœtus which had respired, and one which had not. Great accuracy of observation, and repeated experiments would, however, be requisite, in order to establish the important mark of distinction here laid down by M. P. and after all, were the fact proved, that respiration had taken place, it does not thence necessarily follow that the child was afterwards intentionally destroyed. A chapter containing various directions and remarks respecting the examination of the dead body of an infant, supposed to have been destroyed by violent means, terminates the present volume.

The third and last volume of the present work commences with a chapter on the subject of *Death by Submersion.* Of this article, Dr. De la Fosse is said to be the author. The chief object of inquiry, according to this writer, should be to ascertain, whether the individual had perished in the water, or been thrown into it after death. Here he enters into the consideration of the various proofs and arguments which may be adduced on each side of this question, in order to secure the medical jurist against any erroneous judgment in his conclusions. But, besides the small portion of information conveyed in this paper, we were rather surprised to find, that he had adopted the exploded notion of the reception of water into the bronchia; as likewise, in the subsequent chapter, which treats of *Death by Suspension,* to hear it asserted, by the same author, that in this case life is destroyed by the accession of apoplexy. It is indeed truly astonishing, at the present day, that Dr. De la Fosse appears to be wholly unacquainted with the discovery of Dr. Goodwin, as well as with the labours of succeeding writers in the same tract of inquiry. In this volume we are likewise presented with several specimens of *Medical and Surgical Reports,* which, with Dr. Mahon's observations on this subject, are well deserving the attention of young practitioners. Next succeed some cursory Observations on several questions connected with Legal Medicine, which are followed by a sketch of *Medical Police.* In this division of the work, the regulations necessary to be pursued in lazarettos, hospitals, &c. are considered, and the abuses frequently attendant on such institutions pointed out.

The chapter on Celibacy contains many remarks highly interesting not only to the medical practitioner but to the politician; whilst there are others, in which we can by no means acquiesce, and which could only be tolerated under the most despotic governments.

After considering the state of *Pregnancy,* and that of *Lying-in Women,* in relation to society, on which subjects we find much useful information intermixed with several unimportant remarks, Dr. M. proceeds to treat of the *Cæsarean Operation.*

No question has been more fiercely agitated, in the present day, than the expediency of performing this dreadful operation.

That cases, however, have occurred, in which the narrowness or distortion of the pelvis was so considerable, that the child could not be brought through the natural passages by any art whatever, as well as that the life of both mother and child have been preserved in a few cases by the Cæsarean section, even the most sceptical must, we think, admit upon an impartial examination of the great mass of evidence lately presented by Dr. Hull to the public on this subject. But as the number of cases, in which Hysterotomy has been attended with complete success, is so very inconsiderable, few British practitioners will, we conceive, consider themselves justified in having recourse to to it, without previously attempting every other means of effecting the delivery. Fortunately, the improvements that have been made in the mode of delivery by the crochet, which is now known to be practicable in cases wherein the section of the uterus had formerly been resorted to, warrant us to suppose, that the operation of Embryulcia will in future make the necessity of performing the Cæsarean section an extremely rare occurrence.

Dr. M. however, does not at all enter into the examination of the question, whether the Cæsarean section be preferable to a division of the symphisis pubis, or any other mode of operation in cases of extreme distortion in the pelvis; but merely considers it with a view to the preservation of the child, after the death of the mother. The following are the signs on which, according to him, the performance of this operation should depend.

That the supposed death of the mother shall have been preceded by a severe disease, or by symptoms that usually prove fatal.

That every effort shall have been made to restore the action of the respiratory organs.

That the action of the heart and arterial system shall be no longer perceptible.

That all motion shall have ceased, excepting that of the child in utero.

That the natural heat shall have become extinct, either altogether, or at least in proportion to the continuance of the symptoms which had destroyed the patient; and in the latter case, the heat of the body usually ceases before the last gasp.

Lastly, That all the usual remedies against the different kinds of asphyxia shall have been employed.

Our attention is next called to the subject of *Afflictive Punishments*. Dr. M. like most late writers, reprobates every kind of severity, which is not essentially necessary to public security, and the reformation of the criminal.

The work terminates with a chapter on *Inoculation for the Small-pox*, from which we learn, that our author was a decided advocate in favour of this practice.

Happily, however, the important discovery of Dr. Jenner supersedes the necessity of any discussion respecting that subject, and leads us to indulge the pleasing hope, that, by the application of the vaccine virus to the purpose of inoculation, we may be enabled eventually to extinguish the infection of the small-pox; a disease which has, in every age, proved so destructive to mankind.

From

From the brief Analysis here given, every reader, will, we trust, be enabled to estimate the importance of the present publication, which, as embracing a great variety of topics, and containing much information relative to legal medicine, cannot fail in our opinion, to prove useful not only to the medical practitioner, but also to civilians, and others who require instruction in this department of science.

To the work is prefixed a short biographical sketch of the author.

